# Microplastics in Freshwater Biota: A Critical Review of Isolation, Characterization, and Assessment Methods

**DOI:** 10.1002/gch2.201800118

**Published:** 2019-03-06

**Authors:** James D. O'Connor, Anne Marie Mahon, Anja F. R. M. Ramsperger, Benjamin Trotter, Paula E. Redondo‐Hasselerharm, Albert A. Koelmans, Heather T. Lally, Sinéad Murphy

**Affiliations:** ^1^ Marine and Freshwater Research Centre Department of Natural Science School of Science & Computing Galway‐Mayo Institute of Technology Dublin Road Galway H91 T8NW Ireland; ^2^ Biological Physics Group University of Bayreuth Universitätsstr. 30 95447 Bayreuth Germany; ^3^ Department of Animal Ecology and BayCEER University of Bayreuth Universitätsstr. 30 95447 Bayreuth Germany; ^4^ Aquatic Ecology and Water Quality Management Group Department of Environmental Science Wageningen University & Research Centre P.O. Box 47 6700 AA Wageningen Netherlands

**Keywords:** analytical methods, biomonitoring, invertebrates, plastics

## Abstract

Freshwater systems provide key pathways for microplastic (MP) pollution, and although existing studies have demonstrated the susceptibility of freshwater biota to ingestion, translocation, and trophic transfer, specific challenges pertaining to methodological standardization remain largely unresolved, particularly with respect to isolating, characterizing, and assessing MPs. Here, a critical review is performed outlining the challenges and limitations currently faced by freshwater MP researchers, which may well apply across the MP research spectrum. Recommendations are provided for methodological standardization, particularly in MP characterization, quality assurance, and quality control (QA/QC) procedures as well as reporting. Considerations for the assessment of MPs in freshwater biota as a means of improving comparisons between studies are discussed. Technological advancements, including the improvement of laboratory infrastructure for identifying MPs within the smaller size range as well as methodological standardization are essential in providing policy makers with tools and measures necessary to determine the distribution of MPs within freshwater ecosystems, while also allowing for comparability and providing compliance for future monitoring requirements.

## Graphical abstract

**Current methodologies in the isolation, characterization, and assessment of microplastics (MPs) within freshwater biota** are reviewed. The challenges and limitations currently faced by MP researchers who work in this area are discussed, while recommendations are made for improvements needed to advance this particular field.

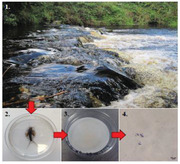

## 1 Introduction

Freshwater systems are closely coupled with the terrestrial environment, and as such, provide key pathways for the influx of basal resources as well as pollutants.^1^, ^2^ Headwater streams serve as important vectors for the transport of materials which may extend into the downstream reaches of rivers, lakes, and estuaries,^3^, ^4^ while longitudinal physical gradients can regulate biotic interactions with such materials by controlling community assemblages.^1^, ^3^ All of which are assumed relevant for microplastic (MP; 1 µm to 5 mm) movement within freshwater food webs. Freshwaters play an important role in the overall lifecycle of microplastics (MPs) in the environment, functioning as receiving waters for waste water discharges and landfill leachates where the majority of MPs are sourced,^5^ while also prolonging the longevity of MPs by retaining them in their sediment^6^, ^7^ and exporting them during high flow or flood events.^8^ Similar to the marine environment, MPs in freshwater are widespread and pervasive, having been reported in the surface waters of lakes^9^, ^10^, ^11^ and rivers,^12^, ^13^, ^14^, ^15^, ^16^ as well as in river and lake shore sediments.^11^, ^17^

Unlike the marine environment however, and until quite recently, MPs in freshwater systems were comparatively understudied,^18^, ^19^, ^20^ with a level of growth in marine scientific literature, in the context of MPs, estimated to be five times that of freshwater ecosystems.^20^ Resultant of which, the hydrodynamics influencing MP behavior in freshwater as well as biological interactions with MPs in natura are relatively undetermined, particularly among lower trophic levels (e.g., primary consumers). The novelty of freshwater MP research, combined with the comparatively low number of field studies that focus on freshwater biota thus far, presents challenges in MP isolation and characterization, especially in methodological standardization, despite the evident parallels with marine MP research and the experimental studies conducted to date. To our knowledge, there are no reviews that focus on the challenges faced in analyzing freshwater biota.

Up to now, most studies on effects in biota were conducted with MP particle concentrations far exceeding those measured in the environment, with an apparent mismatch between particle sizes used to determine effect thresholds in laboratory experimentation and those reported in natura,^21^ something which is difficult to mitigate given the current technological limitations in the verification of polymers collected from the field.^22^ It is noted however that while maintaining ecological relevance is important, what is relevant at present may not be relevant in the future,^23^ and we therefore argue that higher concentrations should also be included to account for future risk.

Here, we present a critical review of the current level of literature pertaining to isolation, characterization, and assessment of MPs in freshwater biota, outlining the challenges and limitations currently faced by freshwater MP researchers, which may well apply to the MP research field as a whole. We provide recommendations for methodological standardization, particularly with regard to particle characterization, quality assurance and quality control (QA/QC) procedures and reporting, as well as considerations for the assessment of MPs in freshwater biota as a means of improving comparisons between studies.

## 2 Microplastics in Freshwater Biota: Occurrence and Methodological Challenges

### 2.1 Microplastics in Freshwater Field Samples

Attention given toward detecting and monitoring MPs in biotic (and abiotic) components of freshwater environments has increased in recent years, expanding our knowledge on susceptibility of species to ingestion,^24^ potential translocation within body tissues,^25^ and trophic transfer of MPs.^26^ Freshwater MP studies undertaken to date on biota have focused on higher taxa, namely fish and birds, with fewer environmental studies assessing lower taxa (**Table** **1**). All studies showed evidence of MP ingestion within the studied species, either through direct consumption and/or secondary ingestion (i.e., trophic transfer). Though for the most part, the specific pathways were not determined. Lower taxa were primarily assessed to ascertain if the prevalence of MPs within individuals, including macroinvertebrates, tadpoles of four species of frog and/or toads, and the Asian clam *Corbicula fluminea* (Müller, 1774), was related to the type (abundance, shape, and polymer distribution) and relative abundance of MPs in water and/or sediment samples, thus assessing their potential as bioindicators of MP pollution in freshwater environments.^26^, ^27^, ^28^, ^29^ Species susceptibility to MP ingestion due to their ecological niche and functional feeding group (grouping feeding habits, traits, and behavior) was also considered in order to assess the relative processes related to the uptake of MPs; processes such as particle selection.^24^, ^26^, ^29^

**Table 1:** Isolation protocols described in field studies reviewed for analyzing MP particles in freshwater biota along with units, shapes, and size ranges

Author(s)	Organism^a)^	Component^b)^	Treatment	Exposure^c)^	Reporting units^d)^	Shapes	Size range(s)
Sanchez et al. 2014^33^	Fish	GI tracts	–	–	–	Focused on fibers	–
Phillips and Bonner 2015^40^	Fish	GI tracts	–	–	–	Films, fragments, and filaments (fibers not recorded)	Max 5.5 mm (linear length)
Faure et al. 2015^9^	Fish and birds	GI tracts	–	–	MP bird^−1^	Mostly fibers and fragments	–
Holland et al. 2016^31^	Birds	GI tracts	–	–	Items bird^−1^	Mostly fragments	50 µm to 5 mm
Peters and Bratton 2016^30^	Fish	GI tracts	–	–	Mean MPs fish^−1^	96% threads (fibrous)	4% > 5 mm (thread size not reported)
Biginagwa et al. 2016^38^	Fish	GI tracts	NaOH (10 mol L^−1^)	24 h (60 °C)	–	–	–
Silva‐Cavalcanti et al. 2017^32^	Fish	GI tracts	–	Particles dried 24 h (70 °C)	No. particles fish^−1^	Fibers only shape described (46.6%)	1–12 mm
Campbell et al. 2017^39^	Fish	GI tracts	10% NaClO and HNO_3_: NaClO (1:10 v/v)	Overnight and until dissolved (room)	Mean MPs fish^−1^	Predominantly fibers and fragments	–
Pazos et al. 2017^34^	Fish	GI tracts	30% H_2_O_2_	Until dissolved (60 °C)	MPs fish^−1^	96% fibers	60 µm to 4.7 mm
Vendel et al. 2017^36^	Fish	GI tracts	–	–	Items total fish^−1^	90% fibers, remainder films and fragments	–
McGoran et al. 2017^37^	Fish	GI tracts	–	–	Mean fibers fish^−1^	Mostly fibers	–
Jabeen et al. 2017^35^	Fish	GI tracts	30% H_2_O_2_ and density separation NaCl (1.2 g cm^−3^)	24–72 h (65 °C)	Items g^−1^, items individual^−1^	Fibers, fragments, and film	40 µm to 5 mm
Horton et al. 2018^41^	Fish	GI tracts	–	–	Particles fish^−1^	Mostly fibers (75%)	<5 mm
Collard et al. 2018^25^	Fish	GI tracts, liver, and muscle tissue	NaClO (14 g L^−1^)	Overnight (room)	AP g^−1^ of stomach content	Fibers dominant	390 µm to 7.38 mm
McNeish et al. 2018^55^	Fish	GI tracts	Dried 24 h (75 °C) 30% H_2_O_2_: Fe(II) (0.05 mol L^−1^)	Until dissolved (75 °C)	No. MPs fish^−1^	Fibers dominant	<1.5 mm, 1.6–3.2 mm, >3.3 mm
Hurley et al. 2017^26^	Invertebrates	Whole	10% KOH	≤10 min (60 °C)	Particles g^−1^ (wet weight)	87% fibers, remainder fragments	55 µm to 4.1 mm
Hu et al. 2018^29^	Tadpoles	Whole	30% H_2_O_2_	≤72 h (65 °C)	Items individual^−1^	Mostly fibers	<0.5 mm
Nel et al. 2018^27^	Invertebrates	Whole	HNO_3_	6 h (room) + 1 h (100 °C)	Particles mg^−1^ (wet weight)	–	–
Su et al. 2018^28^	Molluscs	Soft tissue	30% H_2_O_2_	≤72 h (65 °C)	Items g^−1^	Fibers	0.021–4.02 mm
Windsor et al. 2019^24^	Invertebrates	Whole	Density separation NaCl (1.2 g cm^−3^) and 15% H_2_O_2_	48 h (25°C)	MP mg^−1^	–	500 µm to 5 mm

^a)^Broad classification of organisms studied (invertebrates = benthic macroinvertebrates); ^b)^Component is the target component analyzed for MPs; ^c)^Exposure = time of digestion (temperature); ^d)^Units used to report microplastic burden in terms of abundance or concentration.

Fish and bird species were also assessed as sentinel taxon for MP exposure.^30^, ^31^ Due to the higher trophic level of freshwater fish and the potential for trophic transfer of MPs, most studies have assessed the presence, occurrence, and type of MPs in gastrointestinal (GI) tracts, identifying sampling methodologies for doing so,^32^, ^33^, ^34^, ^35^ some studies, though scant at present, have also analyzed liver and muscle tissue for translocation of MPs,^25^ however potential translocation routes and impacts were not assessed. Studies on fish also included other parameters for assessing the variability in individual MP burdens, including feeding habitats and behaviors (demersal vs pelagic), fish size (assessing consumption and potential bioaccumulation), and the environmental availability of MPs.^30^, ^34^, ^36^, ^37^, ^38^, ^39^, ^40^, ^41^

Feeding behaviors, i.e., whether fish are benthic feeders or pelagic predators, may influence MP ingestion, due to different encounter rates and/or different types of MPs (size, density, color, and morphology) found in the benthic sediment and water column.^36^, ^37^ Trophic roles may also influence MP burdens in fish, where MPs have been consumed via trophic transfer—detritivores and predators may indirectly ingest MPs when consuming prey or scavenging detrital matter.^36^, ^42^

### 2.2 Extracting Microplastics from Biological Samples: Challenges in Isolation

Lack of standardization in isolation methods for MPs internalized in body cavities/tissues or entangled (externally) in biota is currently recognized as a foremost concern across all MP research.^43^, ^44^, ^45^, ^46^ The lack of harmonization across studies means that it is difficult to facilitate comparisons or assess MP exposure levels between similar species.^44^, ^47^ An increasing number of methods have been devised and modified in recent years that include dissection, depuration, homogenization, and digestion of tissue, with the selection of one or more methods largely determined by the research question being explored.^42^ A number of isolation approaches have been observed to damage polymers (e.g., acid digestion),^48^ or under represent smaller MP particles (e.g., visual sorting without digestion).^44^

Post dissection visual observation methods or sorting do not utilize chemicals and are often a preferred method when identifying and separating MPs from freshwater biota, particularly where dietary remains are required to remain intact. Dissection followed by rinsing of GI tracts with water is a well‐practiced protocol in freshwater field studies, especially among fish^9^, ^32^, ^36^, ^37^, ^41^ and birds,^31^ but can lead to an underrepresentation of MPs resultant of challenges with identifying and enumerating smaller particles that may adhere to dietary remains and other biological material within the GI tract of an organism.^31^, ^42^, ^49^ Moreover, the lack of a digestion pretreatment means that where organs are damaged due to freezing or drying out, particle abundance may be underestimated as GI tracts cannot be analyzed in their entirety.^9^

While enzymatic digestion (e.g., trypsin, proteinase‐k)^50^, ^51^ is an emerging isolation technique in MP research, chemical treatments (e.g., oxidizers, acids, bases), due to their less expensive nature and relatively high recovery rates,^52^ are generally the more applied method. Although recently used to extract MPs from freshwater invertebrate samples (i.e., *Chironomus* spp.),^27^ strong mineral acids, such as nitric acid (HNO_3_), have been reported to cause degradation and damage to sensitive polymers (e.g., polyamide (PA), polystyrene (PS), polyethylene (PE))^48^ and are often substituted for bases (e.g., 10% KOH) or oxidizers (e.g., 30% H_2_O_2_) depending on the organ or organism under analysis. Alkaline hydrolysis is easy to perform on soft tissue organisms such as tubificid worms^26^ or on the GI tracts of freshwater fish,^38^ and can be achieved at room temperature.^53^ Wet peroxide oxidation (WPO) using H_2_O_2_, often in the presence of a Fe(II) catalyst (Fenton's reagent), is a preferred method for organisms that contain hard parts such as benthic macroinvertebrates,^24^, ^54^ which usually require an application of heat to break down the chitin within their exoskeleton (personal observation), but has also been used as a method to digest the soft tissue of *C. fluminea*,^28^ tadpoles,^29^ and the GI tracts of fish.^34^, ^35^, ^55^ However such treatments have limitations, with NaOH (10 mol L^−1^) reported to inflict damage or discoloration on PA and PE particles.^49^ Biginagwa et al.^38^ who tested this method prior to digesting the GI tracts of Nile perch *Lates niloticus* (Linnaeus, 1758) and Nile tilapia *Oreochromis niloticus* (L.), report a high digestion efficiency. Similar observations have been made for WPO, which has been found to be an efficient method for breaking down biogenic material despite discoloring (bleaching) MP particles. This could possibly affect the determination of colors rather than leading to an underestimation of particle abundance.^28^, ^56^

### 2.3 Validating Techniques for Microplastic Isolation

As many chemical digestions are known to incur negative effects on the characteristics of certain pH sensitive polymers^48^, ^49^ the choice of digestion method used to isolate MP particles, along with the manner in which it is applied (i.e., heat, level of exposure), can impact significantly upon MP recovery rates.^57^ As well as this, difficulties in determining whether all MPs within a field sample are effectively recovered, renders the validation of such isolation techniques a useful practice within MP research.^45^ Extraction efficacy is typically assessed via “spiking,” whereby MPs of a predetermined abundance, type, size range, and morphology are incorporated into a control sample, subjected to a specific treatment, and the number of MPs recovered expressed as a percentage^58^ (the number of particles remaining in a sample following exposure to an extraction method).^57^ This is generally performed on biological tissue, such as fish,^58^ or wild mussels,^51^ and as recommended by Hermsen et al.,^45^ should be included as both positive samples (i.e., in tissue) and blank samples (negative controls), treated in parallel to field samples.

To our knowledge, there are no freshwater biological studies, from the field at least, that report the testing of such MP isolation protocols, with exception to Biginagwa et al.,^38^ who assessed the digestion efficiency of NaOH in breaking down biogenic material. It is expected however that most biological field studies follow validated techniques. Windsor et al.^24^ applied a method on freshwater benthic macroinvertebrate samples previously tested by Avio et al.,^58^ who report an average extraction yield from laboratory‐acclimatized mullet *Mugil cephalus* (L.) GI tracts of 95% ± 2 (mean% ± SEM), while Hurley et al.^26^ used a 10% KOH solution to digest *Tubifex tubifex* (M.) tissue following a series of protocols previously validated by Karami et al.^59^ on African catfish *Clarias gariepinus* (Burchell, 1822). Some authors, such as Hu et al.,^29^ even replicated extraction methods previously tested by themselves in other studies, whereby a 95% recovery efficiency of spiked microfibers was observed using 30% H_2_O_2_ at temperatures of 65 °C for an exposure period of up to 48 h.^56^ A shortcoming of this however is possible discrepancies in the recovery rates between studies, resultant of the variation in the nature of target components (e.g., soft tissue of GI tracts vs hard macroinvertebrate exoskeleton) and chemicals used.

### 2.4 Considerations for Controlling and Accounting for Microplastic Contamination

The prevalence of contamination in MP research means that QA and QC are paramount throughout all stages of the sample process.^45^, ^60^ As such, rigorous precautions must be adopted while processing field samples, so as to account for any background contamination^44^ and provide accurate data.^60^ The inclusion of procedural blanks, which follow the same analytical protocol,^45^, ^61^ the use of nonplastic tools where possible, as well as natural fiber clothing when sampling and processing^60^ are crucial.

Incidental contamination from airborne particles is a major risk within a MP laboratory, particularly from clothing. While many authors report the use of latex or nitrile gloves and cotton laboratory coats,^9^, ^24^, ^25^, ^37^ only one study from those reviewed,^9^ describe the wearing of natural fiber attire. Hermsen et al.^45^ suggests that the wearing of a 100% cotton laboratory coat alone may not be enough to eradicate contamination should synthetic garments be worn underneath. It is imperative therefore that samples are covered during and between processing steps^61^ so as to keep air exposure to a minimum. In the laboratory, field samples are typically covered with aluminum foil or foil lids^26^, ^34^ but may also be covered with watch glasses.^32^ Some authors also report keeping air contamination to a minimum by working under positive pressure conditions in laminar flow cabinets,^24^, ^39^ which stream filtered air vertically through the work space,^62^ while McGoran et al.^37^ reduced the possibility of airborne contamination by opening fish GI tracts one section at a time.

Due to the high background levels of MPs in the laboratory, particularly microfibers,^63^, ^64^ it is vital that workspaces and tools are inspected and cleaned prior to coming into contact with samples. Many studies report cleaning workspaces and tools with ethanol,^32^, ^36^ some using lint free paper,^41^ as well as bleach mixture (⅓ to ⅔ ratio),^31^ Milli‐Q water,^9^ ultrapure water,^33^ and distilled (DI) water,^25^ while others report using filtered tap water (0.45 µm,^28^ 1 µm^35^). It has been suggested however that the wiping of surfaces with ethanol or water does little in the way of removing particles.^45^ Observing utensils for contamination prior to commencing processing is a recommended practice, and has been detailed by some studies, typically through the use of a stereomicroscope, while Su et al.^28^ also reports storing tools in sealed aluminum bags to keep them clean prior to use. In order to minimize the potential of contamination through the addition of solutions, certain researchers also prefilter liquids (e.g., KOH, NaClO, H_2_O_2_, NaCl) used in sample preparation.^24^, ^25^, ^26^, ^28^, ^29^, ^35^

Procedural blanks are a highly recommended way of accounting for background contamination and isolating steps in the processing stage where contamination can occur.^42^ Many of the field studies reviewed have reported running procedural blanks as part of processing to account for exogenous MP particles.^24^, ^26^, ^28^, ^35^, ^39^, ^55^ Mean MP contamination is generally accounted for based on unique characteristics and may be subtracted from results,^24^, ^55^ or rather, taken into consideration when interpreting results.^28^

## 3 Characterizing Microplastics in Field Studies

### 3.1 The Role of Microplastic Characteristics in Uptake and Trophic Transfer

As evident from the literature reviewed, a considerable number of organisms are exposed to the possibility of MP ingestion. Not only does the morphology of a particle give indication as to the source of MP pollution,^65^ surface characteristics (e.g., polymer, shape, size), combined with organism physiology (e.g., feeding trait and mouth part morphology as well as aperture), may have significant influence on the uptake and residence of MP particles.^46^

Internalization of MP particles has been shown for a variety of freshwater species in laboratory studies including unselective filter feeders like *Daphnia magna* (Straus, 1820)^66^, ^67^, ^68^ and *C. fluminea*,^69^ as well as fish (e.g., zebrafish *Danio rerio* (Hamilton, 1822)).^70^, ^71^ Kolandhasamy et al.^72^ also found a new pathway of MP uptake, albeit in a marine experiment, via adherence of particles to soft tissues of blue mussels *Mytilus edulis* (L.) without being ingested, something which was further demonstrated by Gutow et al.^73^ for the transfer of particles via seaweed to the benthic herbivore *Littorina littorea* (L.). The adherence or entanglement of MPs may therefore play an additional role in the trophic transfer of MP particles through the freshwater food web.

As seen, uptake and prevalence of various polymer types, colors,^26^ and shapes (i.e., fragments, fibers, sheets, and spheres)^9^, ^26^ have been detected in a range of freshwater organisms and are therefore considered valuable criteria when assessing potential pathways of MPs and transfer through aquatic food webs. Particle shape is especially pertinent to residence time, given that specific shapes (i.e., fibers) of 1–5 mm particle length have been shown to aggregate in the gut of certain biota (e.g., crustacea^74^), and is reported in all but three studies reviewed.^24^, ^27^, ^38^ Polymer color is also considered an important descriptor for MP characterization, given that previous research infers a preference for the ingestion of certain colors that potentially resemble prey,^75^, ^76^ and has been described among the majority of freshwater field studies reviewed.^25^, ^26^, ^27^, ^28^, ^31^, ^32^, ^33^, ^34^, ^36^, ^37^, ^39^, ^41^, ^55^ It is noted however that weathering can alter polymer color in the field^76^ as can certain oxidation treatments (e.g., H_2_O_2_) during isolation,^28^ which could possibly result in a certain level of subjectivity in color differentiation during MP characterization.

Therefore, the classification of polymers found in biota in natura should be performed where possible, to further evaluate possible effects of those particles under experimental conditions. It appears however that research groups are often limited, at least from a technological standpoint (e.g., costs, expertise), in the detection or verification of smaller size ranges within field samples.^77^ It should also be noted, that immediately after plastic enters the environment biomolecules interact with them forming an ecocorona on surfaces, changing the plastics properties,^78^, ^79^, ^80^ while a biofilm can also grow on surfaces, affecting ingestion rates among biota.^81^ For example Vroom et al.^81^ showed a higher uptake probability of experimentally aged MPs containing an ecocorona than pristine particles. This indicates that experimental studies using pristine MP particles might underestimate uptake and effects of MPs on biota. Furthermore, as MPs present in the environment undergo transformation processes such as hydrological and UV degradation that influence their surface morphology and behavior,^65^ it is important to prioritize which physical (i.e., surface morphology, size, color), as well as chemical characteristics are ecologically relevant.^46^, ^82^

### 3.2 Characterizing Microplastics in Biota: Size Ranges and Associated Challenges

While laboratory experimental set ups are a good way of ascertaining the relative risk imposed by MPs on an organism, as well as the associated impacts thereof, the physical characteristics of MPs ingested in these scenarios were often observed, as outlined earlier, to be significantly different than those documented in natura.^83^ Additionally, experimental exposure concentrations were generally at least two orders of magnitude higher than those described in the field,^84^ or at best, inclusive of the upper most concentrations reported for freshwater subcompartments (e.g., sediment).^54^

MPs collected in the field are largely characterized by their morphological characteristics such as color, shape, and size, the latter of which is usually measured along the longest axis.^42^ Size categories have been used, but discrepancies in the reporting of particle size ranges among freshwater biota combined with an overall lack of standardization in defining micro‐, meso‐, and macroplastics^20^ renders comparisons between studies, regions, and biota challenging. McNeish et al.^55^ investigating the role of species' trait in MP uptake within riverine fish, categorized MPs into three groups (e.g., small < 1.5 mm, medium 1.6–3.2 mm and large > 3.3 mm), while Hu et al.,^29^ in order to facilitate comparisons in MP ingestion among tadpoles, classified MPs into groups according to the size of the mouth aperture (<0.5, 0.5–1, 1–2, or 2–5 mm). In some cases the size of all particles was deemed to be <5 mm,^41^ while in many other studies it was not recorded,^27^, ^33^, ^36^, ^38^ or simply not reported.^27^ While the verification of smaller size ranges from field samples is desired, a number of authors report polymer identification too difficult with available instruments.^29^, ^37^, ^38^, ^55^ This is not surprising given the current technological limitation in identifying MPs < 20 or < 100 µm with most micro‐Fourier transformed infrared (µFT‐IR) spectrometry systems,^22^ tough recent advances (e.g., µ‐Raman) do allow for polymer verification of particles < 10 µm.^77^, ^85^ Until such systems are readily accessible to research groups however, and the technology is improved (e.g., measurement time), accurate reporting of MPs at the smaller size range, as well as the development of suitable experimental studies looking to incorporate relevant MP characteristics at such size limits remain impeded.

### 3.3 Reporting Microplastics in Environmental Samples

One of the main problems currently within MP research is the reporting of different units for MP quantitation which complicates comparisons between studies.^52^ Within freshwater biotic studies, it is generally the case that the percentage occurrence (%) of MP items is reported, followed by either the number of items ingested per organism,^9^, ^29^, ^30^, ^31^, ^34^, ^35^, ^36^, ^37^, ^39^, ^41^, ^55^ MP mg^−1^ for either dry or wet weight of organism,^24^, ^26^, ^27^, ^28^ or number of items per gram of stomach contents (e.g., in fish)^25^ (see Table 1). Some initial studies do not report MP burden in the context of abundances or concentrations at all however.^33^, ^38^, ^40^ If biota are to be employed as bioindicators of MP pollution within the EU Water Framework Directive and the EU Marine Strategy Framework Directive, units employed should be standardized, at least for comparisons within individual trophic levels. For instance, while it might be feasible for larger consumers (e.g., fish), it is often difficult to report on the number of MPs ingested per individual for smaller organisms (e.g., benthic macroinvertebrates) mainly due to their size and variability thereof, and thus analyzing a subsample comprising of a number of individuals is generally a more practical approach. MP burden in this scenario can be expressed as a concentration of MPs per unit mass (e.g., MPs mg^−1^), which is the preferred unit used among most invertebrate studies reviewed.^24^, ^26^, ^27^, ^28^ The authors concur with previous recommendations for the reporting of additional units,^52^ where possible, to ensure that comparison among studies can be undertaken, at least until such time as a consensus can be reached with regard to MP quantitation. Moreover, the (average) size of particles, as well as the relative abundance of the colors and shape of MPs should be reported.^86^

## 4 Field versus Laboratory Studies: Consistencies and Mismatches

Laboratory based studies describe a wide range of effects of MPs on different taxa including effects on morphology and life history traits, such as reduction in food intake and somatic growth, increased mortality, behavior, or altered enzymatic activity.^67^, ^68^, ^69^, ^70^, ^87^, ^88^, ^89^, ^90^, ^91^, ^92^ However, the lack of observed effects on measured parameters are also described.^54^, ^71^, ^93^, ^94^ When looking at plastic particle concentrations, morphology as well as the polymer types used in experimental studies, a discrepancy between those used and what has been observed in field studies is apparent. Out of the above‐mentioned experimental studies, beads/spheres were the most commonly used morphology type (53%), followed by fragments (35%) and fibers (6%). Whereas field studies assessing MP burden in freshwater biota have reported that fibers are the predominant particle type ingested (ranging from 46.6% to 100%).^28^, ^32^, ^41^ Reasons for the use of spherical beads in experimental studies are multifarious, though being more easily available compared to other morphology types may be a leading factor. As Ogonowski et al.^89^ showed that the effects caused by MPs can be attributed to particle morphology, it is suggested that future experimental studies include particle shapes found in higher concentrations in natura to allow for greater ecological relevance.

Further discrepancies in the reporting of background particle concentrations within field studies and those utilized in experimental studies, as well as the units employed, are apparent. Concentrations from field data are reported as number of particles per surface area (e.g., particles m^−2^) or volume (e.g., particles m^−3^), while, laboratory results in most cases are stated as mass per volume, which makes comparison of concentrations difficult. Particle concentrations in laboratory experiments ranged from environmentally relevant concentrations (such as 400 particles L^−1^) to high concentrations (10^8^ particles L^−1^), though the latter studies were focused on detecting effect‐based thresholds.^94^, ^95^ Whereas field studies have reported concentrations of 0.05 particles m^−3^ to 3.2 × 10^5^ particles m^−3^, with the latter being detected in waste water.^12^, ^14^ This mismatch in the actual concentrations used and units employed has already been addressed by Lenz et al.^84^ for marine systems. The aspect of concentration gains even more importance when one considers that observed effects might not be caused by toxicity of polymers, but either by the sheer number of particles used or by the replacement of nutritious food particles by plastic particles, thus causing a dilution of available food concentration. Animals in aquatic environments are exposed to a diverse array of natural particulate matter. Studies examining the effects by natural particulate matter show similar effects, such as altered feeding rate, survival rate or population growth, which are thought to be caused by MP ingestion.^96^, ^97^ Therefore, interpreting results in order to conclude a reliable and realistic risk assessment of MPs is challenging as most studies did not carry out a particle control with natural particulate matter. The only study we reviewed using such a particle control, such as kaolin or other particulate substances, was implemented by Ogonowski et al.^89^ We therefore suggest the use of naturally occurring suspended solids without a nutritional value as particle controls for pelagic organisms in future studies.

### 4.1 Recommendations for Assessing Microplastics in Biota within Experimental Setups

MP ingestion has been evaluated under laboratory conditions for a variety of freshwater species,^54^, ^89^, ^94^, ^95^, ^98^, ^99^, ^100^ some of which being standard toxicity text species.^101^ As mentioned in previous sections, the use of different approaches makes it difficult to establish clear conclusions on the susceptibility to MP ingestion by aquatic biota and the effects derived from this ingestion.^23^, ^102^, ^103^ Here we summarize the most important factors that need to be taken into account during the assessment of MPs in biota and we propose ways to improve the comparison among studies.

#### 4.1.1 Units

Most studies report the amount of MPs in biota as number of particles (ingested/egested) per organism. However, several studies have suggested that MP ingestion can cause energy depletion due to a lower food assimilation.^54^, ^95^, ^100^, ^104^, ^105^ Therefore, ingestion and egestion can also be presented as the dry weight of MPs/weight of biota using the volume and density of the MPs.^54^ As mentioned earlier, we suggest the use of both units when assessing MPs in biota.

#### 4.1.2 Time

The performance of short‐term ingestion tests can help to screen a wider number of organisms and identify the ones with a higher capacity to ingest MPs. However, while the ingestion of MPs can rapidly occur, the full gut depuration can often take more than 24 h.^99^, ^106^, ^107^ Therefore, laboratory studies should aim at quantifying ingestion and egestion rates over longer exposure times.^108^ Time will also allow for biofilm growth, which can affect the ingestion rates of aquatic biota and renders more ecologically relevant tests.^81^

#### 4.1.3 Concentrations

The use of a wide range of concentrations, including environmentally realistic concentrations but also higher concentrations, can provide information on dose‐dependent effects and ingestion.^23^ In fact, several studies have shown a positive linear relationship between the amount of MPs ingested and present in the surrounding medium (water/sediment).^54^, ^98^ This relationship can be quantified using trophic transfer factors (TTF), which can be used to compare species, MPs or exposure conditions.^109^

#### 4.1.4 Bioavailability

MP ingestion is directly related with the bioavailability of the particles, which depend on MP properties such as size, shape and density, as well as on the species traits and experimental conditions, in particular the exposure medium. When it comes to MP properties, ingestion and effects have been found to be size and shape dependent for several aquatic species.^54^, ^81^, ^98^, ^99^, ^105^, ^110^, ^111^ MP density and biofilm (i.e., the composite particle) will determine whether MPs will be found floating, in the water column or in the sediment, which will also depend on the exposure medium and the exposure time. Laboratory studies ideally should therefore use size, shape and density distributions to determine species ingestion preferences and to maximize environmental realism. In the case of size, the lower and upper size limits could be identified and used to correct for the bioavailable sizes, if detection limits allow.^54^ Species traits such as the feeding behavior, mobility, size, and developmental stage can affect MP bioavailability.^54^, ^94^, ^98^ Therefore, the use of a variety of organisms with different traits can provide information on which species are most susceptible to ingest, and be affected, by MP exposure. Additionally, the environmental conditions will also affect the bioavailability of MPs, as the presence of natural particles such as food or sediment has been found to decrease ingestion.^54^, ^89^, ^98^ For this reason, the addition of natural particles to laboratory systems mimicking environmentally realistic situations is of utmost importance.^103^, ^108^

## 5 Conclusions

While procedures for the isolation of MPs in some higher‐level taxa have been largely established (e.g., GI tracts in fish), little standardization exists for the diverse range of freshwater taxa among lower trophic levels. Standard operating procedures (SOPs) for these processes are needed to optimize extraction efficiencies and increase the representativeness of samples. SOPs could be developed through comparative studies testing a number of digestion techniques on a range of biota that share similar physical characteristics.

It is of equal importance that SOPs for contamination control are implemented. QC/QA measures should include the use of controls (positive and negative), cotton lab coats, reduction or elimination of artificial fibers in the laboratory setting where possible, with further reductions achieved by minimizing air‐exposure times and working in laminar flow cabinets.

While laboratory ingestion experiments have provided valuable information regarding the ingestion of MPs, further improvements are required to improve the efficacy of these tests including increasing running times of ingestion/egestion tests to align with complete gut depuration while allowing for biofouling. Though the incorporation of environmentally relevant MP concentrations is deemed increasingly important in laboratory assessment, higher concentrations are essential for the assessment of dose‐dependent effects and insight into potential future impacts. In any case, the creation of environmentally realistic situations in these studies, including the inclusion of natural particles will provide the optimal outcome.

By using a wide spectrum of MP characteristics during experimentation (i.e., size, shape, polymer type), combined with taxa that exhibit varied physiological traits (e.g., feeding mechanism), factors previously deemed critical for bioavailability can be accounted for. As well as this, standardization of units reported will further enhance comparisons of studies and improve the pertinence of specific measurements (e.g., mass/volume) in effect threshold and feeding studies.

Technological limitation in the identification of MPs within size ranges relevant to lower trophic levels (e.g., macroinvertebrates), as well as lengthy processing times, necessitates improvements in nondestructive and accessible verification methods within MP research (e.g., focal plane array sensors).These technological advances, in addition to methodological standardization are essential in providing policy makers with tools and measures necessary to determine the distribution of MPs in freshwater ecosystems, while allowing comparisons and providing compliance for future monitoring requirements.

## Conflict of Interest

The authors declare no conflict of interest.

## References

[1] G. Woodward , A. G. Hildrew , Freshwater Biol. 2002, 47, 777.

[2] J. S. Baron , N. Poff , Water Resour. Update 2004, 127, 52.

[3] R. L. Vannote , G. W. Minshall , K. W. Cummins , J. R. Sedell , C. E. Cushing , Can. J. Fish. Aquat. Sci. 1980, 37, 130.

[4] W. D. Riley , E. C. Potter , J. Biggs , A. L. Collins , H. P. Jarvie , J. I. Jones , M. Kelly‐Quinn , S. J. Ormerod , D. A. Sear , R. L. Wilby , Sci. Total Environ. 2018, 645, 1598. 30248877 10.1016/j.scitotenv.2018.07.243 PMC6162339

[5] A. M. Mahon , R. Officer , R. Nash , I. O'Connor , Environmental Protection Agency, Wexford, Ireland 2017.

[6] E. Besseling , J. T. Quik , M. Sun , A. A. Koelmans , Environ. Pollut. 2017, 220, 540. 27743792 10.1016/j.envpol.2016.10.001

[7] S. Klein , E. Worch , T. P. Knepper , Environ. Sci. Technol. 2015, 49, 6070. 25901760 10.1021/acs.est.5b00492

[8] R. Hurley , J. Woodward , J. J. Rothwell , Nat. Geosci. 2018, 11, 251.

[9] F. Faure , C. Demars , O. Wieser , M. Kunz , L. F. de Alencastro , Environ. Chem. 2015, 12, 582.

[10] X. Xiong , K. Zhang , X. Chen , H. Shi , Z. Luo , C. Wu , Environ. Pollut. 2018, 235, 899. 29353805 10.1016/j.envpol.2017.12.081

[11] F. Faure , M. Corbaz , H. Baecher , L. de Alencastro , Arch. Sci. 2012, 65, 157.

[12] R. Dris , J. Gasperi , V. Rocher , M. Saad , N. Renault , B. Tassin , Environ. Chem. 2015, 12, 592.

[13] J. Gasperi , R. Dris , T. Bonin , V. Rocher , B. Tassin , Environ. Pollut. 2014, 195, 163. 25240189 10.1016/j.envpol.2014.09.001

[14] A. K. Baldwin , S. R. Corsi , S. A. Mason , Environ. Sci. Technol. 2016, 50, 10377. 27627676 10.1021/acs.est.6b02917

[15] A. McCormick , T. J. Hoellein , S. A. Mason , J. Schluep , J. J. Kelly , Environ. Sci. Technol. 2014, 48, 11863. 25230146 10.1021/es503610r

[16] M. Rodrigues , N. Abrantes , F. Gonçalves , H. Nogueira , J. Marques , A. Gonçalves , Sci. Total Environ. 2018, 633, 1549. 29758905 10.1016/j.scitotenv.2018.03.233

[17] H. K. Imhof , N. P. Ivleva , J. Schmid , R. Niessner , C. Laforsch , Curr. Biol. 2013, 23, R867. 24112978 10.1016/j.cub.2013.09.001

[18] M. Wagner , C. Scherer , D. Alvarez‐Muñoz , N. Brennholt , X. Bourrain , S. Buchinger , E. Fries , C. Grosbois , J. Klasmeier , T. Marti , Environ. Sci. Eur. 2014, 10.1186/s12302-014-0012-7. PMC5566174 28936382

[19] D. Eerkes‐Medrano , R. C. Thompson , D. C. Aldridge , Water Res. 2015, 75, 63. 25746963 10.1016/j.watres.2015.02.012

[20] M. C. Blettler , E. Abrial , F. R. Khan , N. Sivri , L. A. Espinola , Water Res. 2018, 143, 416. 29986250 10.1016/j.watres.2018.06.015

[21] R. Triebskorn , T. Braunbeck , T. Grummt , L. Hanslik , S. Huppertsberg , M. Jekel , T. P. Knepper , S. Krais , Y. K. Müller , M. Pittroff , TrAC Trends Anal. Chem. 2019, 110, 375.

[22] J. Frias , R. Nash , Mar. Pollut. Bull. 2019, 138, 145. 30660255 10.1016/j.marpolbul.2018.11.022

[23] A. A. Koelmans , E. Besseling , E. Foekema , M. Kooi , S. Mintenig , B. C. Ossendorp , P. E. Redondo‐Hasselerharm , A. Verschoor , A. P. Van Wezel , M. Scheffer , Environ. Sci. Technol. 2017, 51, 11513. 28971682 10.1021/acs.est.7b02219 PMC5677762

[24] F. M. Windsor , R. M. Tilley , C. R. Tyler , S. J. Ormerod , Sci. Total Environ. 2019, 646, 68. 30048870 10.1016/j.scitotenv.2018.07.271

[25] F. Collard , J. Gasperi , B. Gilbert , G. Eppe , S. Azimi , V. Rocher , B. Tassin , Sci. Total Environ. 2018, 643, 1257. 30189542 10.1016/j.scitotenv.2018.06.313

[26] R. R. Hurley , J. C. Woodward , J. J. Rothwell , Environ. Sci. Technol. 2017, 51, 12844. 29019399 10.1021/acs.est.7b03567

[27] H. A. Nel , T. Dalu , R. J. Wasserman , Sci. Total Environ. 2018, 612, 950. 28886547 10.1016/j.scitotenv.2017.08.298

[28] L. Su , H. Cai , P. Kolandhasamy , C. Wu , C. M. Rochman , H. Shi , Environ. Pollut. 2018, 234, 347. 29195176 10.1016/j.envpol.2017.11.075

[29] L. Hu , M. Chernick , D. E. Hinton , H. Shi , Environ. Sci. Technol. 2018, 52, 8885. 30035533 10.1021/acs.est.8b02279

[30] C. A. Peters , S. P. Bratton , Environ. Pollut. 2016, 210, 380. 26807984 10.1016/j.envpol.2016.01.018

[31] E. R. Holland , M. L. Mallory , D. Shutler , Sci. Total Environ. 2016, 571, 251. 27476006 10.1016/j.scitotenv.2016.07.158

[32] J. S. Silva‐Cavalcanti , J. D. B. Silva , E. J. de França , M. C. B. de Araújo , F. Gusmão , Environ. Pollut. 2017, 221, 218. 27914860 10.1016/j.envpol.2016.11.068

[33] W. Sanchez , C. Bender , J.‐M. Porcher , Environ. Res. 2014, 128, 98. 24295902 10.1016/j.envres.2013.11.004

[34] R. S. Pazos , T. Maiztegui , D. C. Colautti , A. H. Paracampo , N. Gómez , Mar. Pollut. Bull. 2017, 122, 85. 28633946 10.1016/j.marpolbul.2017.06.007

[35] K. Jabeen , L. Su , J. Li , D. Yang , C. Tong , J. Mu , H. Shi , Environ. Pollut. 2017, 221, 141. 27939629 10.1016/j.envpol.2016.11.055

[36] A. Vendel , F. Bessa , V. Alves , A. Amorim , J. Patrício , A. Palma , Mar. Pollut. Bull. 2017, 117, 448. 28214011 10.1016/j.marpolbul.2017.01.081

[37] A. McGoran , P. Clark , D. Morritt , Environ. Pollut. 2017, 220, 744. 27697381 10.1016/j.envpol.2016.09.078

[38] F. J. Biginagwa , B. S. Mayoma , Y. Shashoua , K. Syberg , F. R. Khan , J. Great Lakes Res. 2016, 42, 146.

[39] S. H. Campbell , P. R. Williamson , B. D. Hall , FACETS 2017, 2, 395.

[40] M. B. Phillips , T. H. Bonner , Mar. Pollut. Bull. 2015, 100, 264. 26388444 10.1016/j.marpolbul.2015.08.041

[41] A. A. Horton , M. D. Jürgens , E. Lahive , P. M. van Bodegom , M. G. Vijver , Environ. Pollut. 2018, 236, 188. 29414339 10.1016/j.envpol.2018.01.044

[42] A. Lusher , N. Welden , P. Sobral , M. Cole , Anal. Methods 2017, 9, 1346.

[43] S. Rezania , J. Park , M. F. M. Din , S. M. Taib , A. Talaiekhozani , K. K. Yadav , H. Kamyab , Mar. Pollut. Bull. 2018, 133, 191. 30041307 10.1016/j.marpolbul.2018.05.022

[44] G. Vandermeersch , L. Van Cauwenberghe , C. R. Janssen , A. Marques , K. Granby , G. Fait , M. J. Kotterman , J. Diogène , K. Bekaert , J. Robbens , Environ. Res. 2015, 143, 46. 26249746 10.1016/j.envres.2015.07.016

[45] E. Hermsen , S. M. Mintenig , E. Besseling , A. A. Koelmans , Environ. Sci. Technol. 2018, 52, 10230. 30137965 10.1021/acs.est.8b01611 PMC6146318

[46] S. Y. Au , C. M. Lee , J. E. Weinstein , P. van den Hurk , S. J. Klaine , Integrated Environ. Assess. Manage. 2017, 13, 505. 10.1002/ieam.1907 28440939

[47] A. I. Catarino , R. Thompson , W. Sanderson , T. B. Henry , Environ. Toxicol. Chem. 2017, 36, 947. 27583696 10.1002/etc.3608

[48] M. Claessens , L. Van Cauwenberghe , M. B. Vandegehuchte , C. R. Janssen , Mar. Pollut. Bull. 2013, 70, 227. 23601693 10.1016/j.marpolbul.2013.03.009

[49] M. Cole , H. Webb , P. K. Lindeque , E. S. Fileman , C. Halsband , T. S. Galloway , Sci. Rep. 2014, 10.1038/srep04528. PMC3970126 24681661

[50] M. G. Löder , H. K. Imhof , M. Ladehoff , L. A. Löschel , C. Lorenz , S. Mintenig , S. Piehl , S. Primpke , I. Schrank , C. Laforsch , Environ. Sci. Technol. 2017, 51, 14283. 29110472 10.1021/acs.est.7b03055

[51] W. Courtene‐Jones , B. Quinn , F. Murphy , S. F. Gary , B. E. Narayanaswamy , Anal. Methods 2017, 9, 1437.

[52] S. Klein , I. K. Dimzon , J. Eubeler , T. P. Knepper , in The Handbook of Environmental Chemistry, Vol. 58 (Eds: M. Wagner , S. Lambert ), Springer, Cham, Switzerland 2018, pp. 51–67.

[53] S. Kühn , B. Van Werven , A. Van Oyen , A. Meijboom , E. L. B. Rebolledo , J. A. Van Franeker , Mar. Pollut. Bull. 2017, 115, 86. 27912918 10.1016/j.marpolbul.2016.11.034

[54] P. E. Redondo‐Hasselerharm , D. Falahudin , E. T. Peeters , A. A. Koelmans , Environ. Sci. Technol. 2018, 52, 2278. 29337537 10.1021/acs.est.7b05367 PMC5822217

[55] R. McNeish , L. Kim , H. Barrett , S. Mason , J. Kelly , T. Hoellein , Sci. Rep. 2018, 8, 1. 30076314 10.1038/s41598-018-29980-9 PMC6076259

[56] J. Li , X. Qu , L. Su , W. Zhang , D. Yang , P. Kolandhasamy , D. Li , H. Shi , Environ. Pollut. 2016, 214, 177. 27086073 10.1016/j.envpol.2016.04.012

[57] K. Munno , P. A. Helm , D. A. Jackson , C. Rochman , A. Sims , Environ. Toxicol. Chem. 2018, 37, 91. 28782833 10.1002/etc.3935

[58] C. G. Avio , S. Gorbi , F. Regoli , Mar. Environ. Res. 2015, 111, 18. 26210759 10.1016/j.marenvres.2015.06.014

[59] A. Karami , A. Golieskardi , C. K. Choo , N. Romano , Y. B. Ho , B. Salamatinia , Sci. Total Environ. 2017, 578, 485. 27836345 10.1016/j.scitotenv.2016.10.213

[60] L. Mai , L.‐J. Bao , L. Shi , C. S. Wong , E. Y. Zeng , Environ. Sci. Pollut. Res. 2018, 25, 11319. 10.1007/s11356-018-1692-0 29536421

[61] R. Dris , H. Imhof , W. Sanchez , J. Gasperi , F. Galgani , B. Tassin , C. Laforsch , Environ. Chem. 2015, 12, 539.

[62] C. Wesch , A. M. Elert , M. Wörner , U. Braun , R. Klein , M. Paulus , Sci. Rep. 2017, 10.1038/s41598-017-05838-4. PMC5511265 28710404

[63] L. C. Woodall , C. Gwinnett , M. Packer , R. C. Thompson , L. F. Robinson , G. L. Paterson , Mar. Pollut. Bull. 2015, 95, 40. 25936572 10.1016/j.marpolbul.2015.04.044

[64] M.‐T. Nuelle , J. H. Dekiff , D. Remy , E. Fries , Environ. Pollut. 2014, 184, 161. 24051349 10.1016/j.envpol.2013.07.027

[65] S. Rist , N. B. Hartmann , in The Handbook of Environmental Chemistry, Vol. 58 (Eds: M. Wagner , S. Lambert ), Springer, Cham, Switzerland 2018, pp. 25–49.

[66] A. Jemec , P. Horvat , U. Kunej , M. Bele , A. Kržan , Environ. Pollut. 2016, 219, 201. 27814536 10.1016/j.envpol.2016.10.037

[67] S. Rehse , W. Kloas , C. Zarfl , Chemosphere 2016, 153, 91. 27010171 10.1016/j.chemosphere.2016.02.133

[68] A. Martins , L. Guilhermino , Sci. Total Environ. 2018, 631–632, 421. 10.1016/j.scitotenv.2018.03.054 29529430

[69] L. Guilhermino , L. R. Vieira , D. Ribeiro , A. S. Tavares , V. Cardoso , A. Alves , J. M. Almeida , Sci. Total Environ. 2018, 622–623, 1131. 10.1016/j.scitotenv.2017.12.020 29890582

[70] L. Lei , S. Wu , S. Lu , M. Liu , Y. Song , Z. Fu , H. Shi , K. M. Raley‐Susman , D. He , Sci. Total Environ. 2018, 619–620, 1. 10.1016/j.scitotenv.2017.11.103 29136530

[71] S. Rainieri , N. Conlledo , B. K. Larsen , K. Granby , A. Barranco , Environ. Res. 2018, 162, 135. 29306661 10.1016/j.envres.2017.12.019

[72] P. Kolandhasamy , L. Su , J. Li , X. Qu , K. Jabeen , H. Shi , Sci. Total Environ. 2018, 610–611, 635. 10.1016/j.scitotenv.2017.08.053 28822931

[73] L. Gutow , A. Eckerlebe , L. Giménez , R. Saborowski , Environ. Sci. Technol. 2016, 50, 915. 26654910 10.1021/acs.est.5b02431

[74] A. J. Watts , M. A. Urbina , S. Corr , C. Lewis , T. S. Galloway , Environ. Sci. Technol. 2015, 49, 14597. 26529464 10.1021/acs.est.5b04026

[75] S. L. Wright , R. C. Thompson , T. S. Galloway , Environ. Pollut. 2013, 178, 483. 23545014 10.1016/j.envpol.2013.02.031

[76] C. Wu , K. Zhang , X. Xiong , in The Handbook of Environmental Chemistry, Vol. 58 (Eds: M. Wagner , S. Lambert ), Springer, Cham, Switzerland 2018, pp. 85–99.

[77] J. Li , H. Liu , J. P. Chen , Water Res. 2018, 137, 362. 29580559 10.1016/j.watres.2017.12.056

[78] T. S. Galloway , M. Cole , C. Lewis , Nat. Ecol. Evol. 2017, 10.1038/s41559-017-0116. 28812686

[79] M. Lundqvist , J. Stigler , T. Cedervall , T. Berggård , M. B. Flanagan , I. Lynch , G. Elia , K. Dawson , ACS Nano 2011, 5, 7503. 21861491 10.1021/nn202458g

[80] M. Markiewicz , J. Kumirska , I. Lynch , M. Matzke , J. Köser , S. Bemowsky , D. Docter , R. Stauber , D. Westmeier , S. Stolte , Green Chem. 2018, 20, 4133.

[81] R. J. Vroom , A. A. Koelmans , E. Besseling , C. Halsband , Environ. Pollut. 2017, 231, 987. 28898955 10.1016/j.envpol.2017.08.088

[82] C. Scherer , A. Weber , S. Lambert , M. Wagner , in The Handbook of Environmental Chemistry, Vol. 58 (Eds: M. Wagner , S. Lambert ), Springer, Cham, Switzerland 2018, pp. 153–180.

[83] X. Qu , L. Su , H. Li , M. Liang , H. Shi , Sci. Total Environ. 2018, 621, 679. 29197287 10.1016/j.scitotenv.2017.11.284

[84] R. Lenz , K. Enders , T. G. Nielsen , Proc. Natl. Acad. Sci. USA 2016, 113, E4121. 27407153 10.1073/pnas.1606615113 PMC4961204

[85] C. F. Araujo , M. M. Nolasco , A. M. Ribeiro , P. J. Ribeiro‐Claro , Water Res. 2018, 142, 426. 29909221 10.1016/j.watres.2018.05.060

[86] J. Gago , F. Galgani , T. Maes , R. C. Thompson , Front. Mar. Sci. 2016, 10.3389/fmars.2016.00219.

[87] L. G. A. Barboza , L. R. Vieira , L. Guilhermino , Environ. Pollut. 2018, 236, 1014. 29449115 10.1016/j.envpol.2017.12.082

[88] F. Murphy , B. Quinn , Environ. Pollut. 2018, 234, 487. 29216486 10.1016/j.envpol.2017.11.029

[89] M. Ogonowski , C. Schür , Å. Jarsén , E. Gorokhova , PLoS One 2016, 11, e0155063. 27176452 10.1371/journal.pone.0155063 PMC4866784

[90] S. Rist , A. Baun , N. B. Hartmann , Environ. Pollut. 2017, 228, 398. 28554029 10.1016/j.envpol.2017.05.048

[91] P. Yu , Z. Liu , D. Wu , M. Chen , W. Lv , Y. Zhao , Aquat. Toxicol. 2018, 200, 28. 29709883 10.1016/j.aquatox.2018.04.015

[92] F. Lagarde , O. Olivier , M. Zanella , P. Daniel , S. Hiard , A. Caruso , Environ. Pollut. 2016, 215, 331. 27236494 10.1016/j.envpol.2016.05.006

[93] S. B. Sjollema , P. Redondo‐Hasselerharm , H. A. Leslie , M. H. Kraak , A. D. Vethaak , Aquat. Toxicol. 2016, 170, 259. 26675372 10.1016/j.aquatox.2015.12.002

[94] A. Weber , C. Scherer , N. Brennholt , G. Reifferscheid , M. Wagner , Environ. Pollut. 2018, 234, 181. 29175683 10.1016/j.envpol.2017.11.014

[95] S. Y. Au , T. F. Bruce , W. C. Bridges , S. J. Klaine , Environ. Toxicol. Chem. 2015, 34, 2564. 26042578 10.1002/etc.3093

[96] J. M. Chapman , C. L. Proulx , M. A. Veilleux , C. Levert , S. Bliss , M.‐E. Andre , N. W. Lapointe , S. J. Cooke , Water Res. 2014, 56, 190. 24681235 10.1016/j.watres.2014.02.047

[97] M. E. Kjelland , C. M. Woodley , T. M. Swannack , D. L. Smith , Environ. Syst. Decisions 2015, 35, 334.

[98] C. Scherer , N. Brennholt , G. Reifferscheid , M. Wagner , Sci. Rep. 2017, 10.1038/s41598-017-17191-7. PMC5717137 29208925

[99] S. Grigorakis , S. A. Mason , K. G. Drouillard , Chemosphere 2017, 169, 233. 27880921 10.1016/j.chemosphere.2016.11.055

[100] S. Straub , P. E. Hirsch , P. Burkhardt‐Holm , Int. J. Environ. Res. Public Health 2017, 14, 774. 10.3390/ijerph14070774 PMC5551212 28703776

[101] C. M. Rochman , Science 2018, 360, 28. 29622640 10.1126/science.aar7734

[102] A. Karami , Chemosphere 2017, 184, 841. 28646766 10.1016/j.chemosphere.2017.06.048

[103] K. A. Connors , S. D. Dyer , S. E. Belanger , Environ. Toxicol. Chem. 2017, 36, 1697. 28543985 10.1002/etc.3829

[104] S. L. Wright , D. Rowe , R. C. Thompson , T. S. Galloway , Curr. Biol. 2013, 23, R1031. 24309274 10.1016/j.cub.2013.10.068

[105] S. Ziajahromi , A. Kumar , P. A. Neale , F. D. Leusch , Environ. Pollut. 2018, 236, 425. 29414367 10.1016/j.envpol.2018.01.094

[106] D. Mazurais , B. Ernande , P. Quazuguel , A. Severe , C. Huelvan , L. Madec , O. Mouchel , P. Soudant , J. Robbens , A. Huvet , Mar. Environ. Res. 2015, 112, 78. 26412109 10.1016/j.marenvres.2015.09.009

[107] E. M. Chua , J. Shimeta , D. Nugegoda , P. D. Morrison , B. O. Clarke , Environ. Sci. Technol. 2014, 48, 8127. 24884099 10.1021/es405717z

[108] E. E. Burns , A. B. Boxall , Environ. Toxicol. Chem. 2018, 37, 2776. 30328173 10.1002/etc.4268

[109] D. K. DeForest , K. V. Brix , W. J. Adams , Aquat. Toxicol. 2007, 84, 236. 17673306 10.1016/j.aquatox.2007.02.022

[110] K.‐W. Lee , W. J. Shim , O. Y. Kwon , J.‐H. Kang , Environ. Sci. Technol. 2013, 47, 11278. 23988225 10.1021/es401932b

[111] A. D. Gray , J. E. Weinstein , Environ. Toxicol. Chem. 2017, 36, 3074. 28594093 10.1002/etc.3881

